# Residential care status and gender differences in executive memory and depressive symptoms among older adults

**DOI:** 10.1093/geroni/igaf122.2463

**Published:** 2025-12-31

**Authors:** Idorenyin Udoh, Rie Suzuki

**Affiliations:** University of Michigan Flint, Flint, Michigan, United States; University of Michigan-Flint, Flint, Michigan, United States

## Abstract

**Background:**

Older adults often experience declining memory and feelings of depression. This can further vary based on differences in their residential care status and gender. The purpose of this study is to examine if gender and residential care status differentiate in memory and depression among older adults so that caregivers and healthcare providers can tailor interventions to the specific needs of older adults.

**Methods:**

Data was analyzed from the National Health and Aging Trends Data set round 12, consisting of 6327 participants. Independent variables were residential location (community and residential facilities) and gender. Dependent variables were executive memory and depressive symptoms. Weighted descriptive statistics and two-way ANOVAs with post hoc tests were utilized.

**Results:**

Most participants were female (58.1%) and community residents (87.1%) aged between 65 and above 90 years. About 47.6% of participants reported executive memory issues, and 53.1% reported depressive symptoms. The results showed that males in residential facilities independently showed better executive memory function [F (1, 3006) = 4.446, p = .035]. The interaction between gender and residential location on executive memory function indicated that, compared to females, males living in residential care facilities showed better executive memory (p < .001). However, the direct and interaction effects did not show any mean differences in the presence of depressive symptoms.

**Conclusion:**

Findings highlight the importance of considering residential status and gender when designing interventions and support systems for older adults, enabling healthcare providers to offer more personalized and effective care.

